# Caught in the Web of the Net? Part I: Meta-analyses of Problematic Internet Use and Social Media Use in (Young) People with Autism Spectrum Disorder

**DOI:** 10.1007/s10567-025-00524-8

**Published:** 2025-04-23

**Authors:** Peter Muris, Henry Otgaar, Franc Donkers, Thomas H. Ollendick, Anne Deckers

**Affiliations:** 1 Department of Clinical Psychological Science, Faculty of Psychology and Neuroscience, Maastricht University, Maastricht, The Netherlands; 2 Stellenbosch University, Stellenbosch, South Africa; 3 Youz-Parnassia Group, Maastricht, The Netherlands; 4 Catholic University Leuven, Leuven, Belgium; 5 Virginia Polytechnic Institute and State University, Blacksburg, VA USA; 6 Zuyderland Medisch Centrum, Heerlen, The Netherlands

**Keywords:** Autism spectrum disorder (ASD), Problematic internet use (PIU), Social media use, Meta-analysis

## Abstract

This article examined the internet and social media usage among (young) individuals with autism spectrum disorder (ASD). Two meta-analyses were conducted to quantify (1) the relation between ASD/autistic traits and problematic internet use (PIU, which included generalized PIU, problematic gaming, excessive smartphone use), and (2) the relation between ASD/autistic traits and social media use. The results of our first meta-analysis—comprising 46 studies and 42,274 participants—revealed that people with ASD or higher levels of autistic traits showed higher levels of PIU, with an average effect size of *r* = 0.26 (95% CI [0.21, 0.31]). The second meta-analysis—consisting of 15 studies and 7036 participants—indicated that people with ASD or higher levels of autistic traits were less involved on social media platforms as compared to their typically developing counterparts, with the average effect size being *r* = − 0.28 (95% CI [− 0.38, − 0.18]). The quality of the research on PIU and social media in persons with ASD was critically evaluated and possible directions for future research on this topic are discussed.

## Introduction

Internet-based media have become an indispensable part of contemporary life. Although there are still substantial differences across low and high-income countries, globally the majority of all adults nowadays own a computer, smartphone, and/or tablet that they can use to go online for educational, occupational, social, and recreational purposes (https://www.pewresearch.org). The digitalization of society has not bypassed its younger members. In fact, the evidence indicates that young people typically start to use screen-based media during the first decade of life and spend a substantial amount of time on internet-mediated activities (e.g., Reid Chassiakos et al., 2016). To illustrate this point, Odgers and Jensen (2020) reported data of a study conducted in the United States of America showing that children aged 8 to 12 years already spent on average almost 5 h per day on online screen-based activities, whereas in older youths aged 13 to 18 years the time devoted to internet-mediated media was even higher and approaching an average of seven hours per day (Rideout, 2015). Although collected a decade ago, it is safe to assume that these figures have not decreased among more recent generations of children and adolescents (https://www.pewresearch.org).

While it has been argued that the use of digital media is associated with potential benefits such as the acquisition of knowledge, the initiation and maintenance of social relationships and friendships, and the development of specific cognitive skills, screen-based activities may simultaneously pose risks for young people’s well-being and development, such as exposure to age-inappropriate (violent or sexual) content, experiences of cyberbullying, distraction from schoolwork, and less engagement in physical activities (Canadian Paediatric Society Digital Health Task Force, 2017). These hazards increase when children and adolescents are excessively involved with digital devices and/or spend so much time on internet-mediated activities that it interferes with their daily functioning (Muppalla et al., 2023).

In the psychological literature, the label ‘internet addiction’ has been used to define a level of internet use that is out of control and as such has a significant impact on personal life (Shaw & Black, 2008). Instruments used to assess this type of problem—such as Young’s (1998) Internet Addiction Test—commonly discern the prototypical characteristics of this problem, including an excessive amount of time spent on online activities (e.g., “Do others in your life complain to you about the amount of time you spend online?”), an emotional and cognitive preoccupation with the internet (e.g., “Do you feel depressed, moody, or nervous when you are offline?”, “Do you keep thinking about the previous or next online session?”), a loss of control (e.g., “Do you find that you stay longer online than intended?”), and interference with daily functioning (e.g., “Does your (school)work suffer because of the amount of time you spend online?”; Moon et al., 2018). Although the excessive, uncontrolled, and dysfunctional use of the internet certainly has ‘addictive’ features, various scholars have questioned whether the phenomenon should be considered as a true addiction (Caplan, 2002; Weinstein & Lejoyeux, 2010; Yellowlees & Marks, 2007) and therefore prefer the term problematic internet use (PIU), which can be regarded as less dysfunctional (Fernandes et al., 2019).

The prevalence of PIU is quite high. A recent meta-analysis by Pan et al. (2020), which was based on 113 epidemiological studies published between 1996 and 2018 including almost 700,000 participants from 31 countries across the world, found an average prevalence rate of 7%. Interestingly, an additional analysis indicated that the prevalence rates were moderated by year of publication: higher rates of PIU were obtained in studies that were published at a later point-in-time. This indicates that the occurrence of this problem has increased over the past decades and additional analyses have revealed that this conclusion is particularly true for the younger generations (Lozano-Blasco et al., 2022a, 2022b). Although PIU has a significant impact on people’s quality of life and its severity can certainly take on pathological proportions (Noroozi et al., 2021), the problem often remains undetected in clinical practice (Kuss & Lopez-Fernandez, 2016). An important reason for this might be the fact that PIU is no official diagnosis in current classification systems such as the Diagnostic and Statistical Manual of Mental Disorders (American Psychiatric Association, 2022) and the International Classification of Diseases (World Health Organization, 2019; see Weinstein & Lejoyeux, 2010). The reason is because there is still debate on whether this problem should be seen as a separate diagnostic entity or as a comorbid phenomenon of other psychiatric disorders (Moretta et al., 2022).

Regarding the latter, research has consistently demonstrated that there are three specific disorders that share a positive relationship with PIU, namely social anxiety disorder (Ding et al., 2023; Prizant-Passal et al., 2016), depression (Gu et al., 2024; Ye et al., 2023), and attention-deficit hyperactivity disorder (ADHD; Wang et al., 2017; Werling et al., 2022). It is good to keep in mind that the relations between PIU and these psychopathologies are likely to be bidirectional in nature (Carli et al., 2013). Thus, the presence of social anxiety disorder, depression, and ADHD may increase the risk for developing PIU, but vice versa it is also plausible that excessive engagement in online activities contributes to the maintenance and expression of the problems associated with these psychiatric conditions. Furthermore, different mechanisms are thought to be operating in each of these disorders. That is, in social anxiety disorder, the internet can be seen as avoidance behavior: the engagement in online interactions is sometimes a way to evade the feelings of apprehension associated with real-life communication (Lee & Stapinski, 2012). In depression, the internet would offer plenty of opportunities to deal with feelings of sadness and loneliness (Gu et al., 2024), while in ADHD, the internet prompts the (understimulated) reward system (Weinstein & Lejoyeux, 2020).

ASD is another plausible psychiatric condition that might be associated with PIU. ASD is considered a developmental disorder characterized by atypical brain development and functioning (Lord et al., 2020) and manifests itself in two prototypical characteristics: (A) *persistent deficits in social communication and social interaction*, which are concerned with shortcomings in social-emotional reciprocity, use of non-verbal communication behaviors, and the development of interpersonal relationships; and (B) *repetitive and restrictive behaviors and interests (RRBIs)*, which pertain to stereotyped and repetitive speech or motor movements, strong adherence of routines and rituals, intense and fixated interests, and specific sensory sensitivities (Hirota & King, 2023). In contemporary psychiatric classification systems, ASD is seen as a dimensional condition, with high levels of autistic characteristics defining the more severe, clinical cases on the autism spectrum (American Psychiatric Association, 2022), which have a prevalence rate of 0.76% (Baxter et al., 2015). However, autism traits also occur at lower levels in the general population, in which subclinical cases of autism can be identified (Ruzich et al., 2015). Regarding the use of digital media and the internet, various scholars noted that people with ASD are generally strongly attracted to screens (Ophir et al., 2023; Slobodin et al., 2019; Westby, 2021) and view internet-based media as a highly preferred leisure activity (Stiller & Mößle, 2018). Many online activities require less use of social skills, are instantly rewarding, stimulate the sensory modalities, offer an escape from reality, and satisfy specific interests, which are all features that make them highly attractive for people with ASD (Durkin, 2010) thereby possibly increasing their risk for developing PIU.

The empirical investigation of the relation between ASD/autistic traits and PIU began more than a decade ago. The studies conducted so far can be roughly divided into three main categories. The first category pertains to studies that compared the prevalence of PIU between a group of individuals with ASD and a control group of individuals without ASD. The second category is concerned with investigations that evaluated the level of ASD (symptoms) or autistic traits in groups of participants with and without (a form of) internet addiction. The third and final category is based on the dimensional nature of autism and simply assesses ASD-related traits and symptoms of PIU in the general or clinical population to examine the (positive) relationship between these two constructs. In recent years, reviews on this topic have appeared that provide an overview of the main findings collected so far. The reviews by Craig et al. (2021) and Normand et al. (2022) mainly focused on the first category of studies, generally demonstrating that children, adolescents, and adults with ASD are at greater risk for gaming disorder and PIU in general than control groups of individuals without ASD. The systematic review by Murray et al. (2022a) was more inclusive and included 21 studies from all three categories. Their conclusion was highly similar: the majority of investigations found positive associations between autistic traits and PIU and between ASD and (clinical manifestations of) internet addiction, and—although they did not conduct a formal meta-analysis—their impression was that the effect sizes of the observed relations were quite variable and mostly in the small to moderate range. The most recent and also most comprehensive analysis of the literature was conducted by Eltahir et al. (2025). Their systematic review, which included 31 studies on gaming disorder or internet addiction, showed that “these conditions appear to be overrepresented in autistic populations” (p. 1), with some studies reporting PIU frequency rates exceeding 20%.

The main purpose of our scientific endeavor was to further explore and understand the internet use of (young) people with ASD. Part I of our venture, which is reported in the present article, aimed to examine the relations between ASD/autistic traits and various types of internet use adopting a meta-analytic approach. In part II (Muris et al., 2025), we adopted a more theoretical perspective and propose a motivation-driven developmental psychopathology model that explains the idiosyncrasies in the use of internet-mediated digital media of people with ASD.

The first objective of the study presented in Part I was to substantiate and quantify the observations offered in the qualitative reviews by Craig et al. (2021), Eltahir et al. (2025), Murray et al. (2022a), and Normand et al. (2022) by carrying out a meta-analysis of studies examining the relationship between ASD and autistic traits, on the one hand, and PIU, on the other hand. A search was conducted in the extant literature to identify studies that examined the relation between ASD/autistic traits and various types of PIU (i.e., generalized PIU, gaming addiction, and smartphone addiction). In keeping with the results of aforementioned reviews, we hypothesized a positive association between ASD/autistic traits and PIU. We also expected findings to be quite heterogeneous (Murray et al., 2022a), and explored a number of possible moderators that could account for this variation in observed effects, including type of PIU, age group (children/adolescents, young adults, adults), study method (groupwise comparisons of ASD and non-ASD or addicted and non-addicted participants, correlational design), and sample characteristics (clinical versus non-clinical populations).

Furthermore, we conducted an additional meta-analysis on the use of social media in individuals with ASD. Social media refers to online platforms and applications designed to connect people by enabling interaction, communication, and the sharing of personal information (Aichner et al., 2021). Because ASD is typically associated with social communication and interaction difficulties in real life, this special class of internet-based media might offer a viable alternative to relate to other people and engage in interpersonal relationships. This would be in line with the notion that individuals with ASD also have a fundamental ‘need to belong’ (Baumeister & Leary, 1995; e.g., Deckers et al., 2014) and that this could be satisfied through the use of social media (Leung et al., 2023; Parent, 2023). In the meantime, it remains unclear to what extent people with ASD display similar levels of social media use as their typically developing counterparts. According to the social motivation theory of autism (Chevallier et al., 2012), individuals with ASD have less drive to interact and communicate with others and therefore might show less interest and involvement in social media. Furthermore, it has also been noted that persons high on the spectrum sometimes struggle with the ‘social’ aspects of social media, advancing another argument for why they would make less use of the typical social online platforms (Wang et al., 2020).

## Method

Two searches of the extant literature were conducted in the Web-of-Science database. The first search was carried out between February 23, 2024 and March 5, 2024, and focused on retrieving studies that in some way examined the relationship between ASD/autistic traits and PIU. For ASD/autistic traits, the search terms were *autism*, *autistic*, *pervasive developmental disorder*, or *Asperger* (in topic), while for PIU the terms *internet*, *smartphone*, *gaming*, *screen*, and *digital media* (in topic) were systematically combined with *addiction*, *excessive*, *problematic*, *compulsive*, and *disorder* (in topic). For each identified article, the title and abstract were carefully read to determine whether it: (1) concerned an empirical study, (2) was written in English, (3) was published in a peer-reviewed journal, and (4) possibly contained data on the relation between ASD/autistic traits and PIU. Following this, potentially relevant articles were thoroughly read and only considered eligible when: (5) one of the following study methods was employed: (a) an ASD versus control group comparison with regard to the level of PIU; (b) a PIU versus non-PIU group comparison with regard to the level of autistic traits or presence of an ASD diagnosis; or (c) a correlational analysis of the link between autistic traits and symptoms of PIU. Research that fulfilled all these inclusion criteria became part of the meta-analyses, but studies that for example only presented qualitative data (case descriptions) or a review, or that included no comparison group and/or no measurement(s) of ASD/autistic traits or PIU were all excluded. In the first week of January 2025, the search was repeated (using the same search terms and inclusion criteria) to detect new relevant studies that had appeared in 2024.

From each of the selected articles, the following details were extracted: study and sample characteristics (i.e., authors plus year of publication, sample size, type of population [i.e., clinical, non-clinical] and developmental groups [children and adolescents, young adults, and adults], age range/mean age, and country where the research was conducted), the study method (i.e., comparison ASD versus non-ASD groups, comparison PIU versus non-PIU groups, or correlational), the type of PIU under investigation (i.e., generalized PIU, problematic gaming, excessive smartphone use), and in what way ASD/autistic traits and PIU were measured. Effect sizes were expressed as *r* (correlation coefficient), because the statistics used to examine the relationship between ASD/autistic traits and PIU were variable across studies, and this index can be derived from various types of data (e.g., means and standard deviations of two groups, two (ASD versus control) by two frequencies (PIU versus no PIU), and correlation with sample size). Wilson’s (2023) online effect size calculator (https://www.campbellcollaboration.org) was used to calculate Fisher’s *Z*-transformed *r* and the accompanying confidence interval (CI) for each study. In case multiple *r*’s were obtained from one and the same study, the Fisher’s Z *r*’s and the accompanying confidence interval (CI) were averaged. JAMOVI software (https://www.jamovi.org) was used to combine the transformed *r*’s and CI’s, to estimate the meta-analysis model using the restricted Maximum Likelihood method, compute heterogeneity statistics, and to obtain forest and funnel plots. We expected to document positive effect sizes, indicating that the presence of ASD or high levels of autistic traits are associated with higher levels of PIU (and vice versa). To interpret the obtained effect sizes, we used Funder and Ozer’s (2019) criteria (which define *r*’s < 0.05 as ‘tiny’, *r*’s between 0.05 and 0.10 as ‘very small’, *r*’s between 0.10 and 0.20 as ‘small’, *r*’s between 0.20 and 0.30 as ‘medium’, *r*’s between 0.30 and 0.40 as ‘large’, and *r*’s > 0.40 as ‘very large’) as well as their binominal effect size display.

The second search was conducted on July 8, 2024, and aimed at finding studies that investigated the relationship between ASD/autistic traits and social media use. The search terms were *autism*, *autistic*, *pervasive developmental disorder*, or *Asperger* (in topic) for ASD/autistic traits, in combination with *social media* (in topic). The basic inclusion criteria for eligible articles were that the study was (1) empirical in nature, (2) written in English, (3) published in a peer-reviewed journal, and (4) reported data on the relation between ASD/autistic traits and the use of social media. Regarding the latter, the following study methods were considered relevant: (a) an ASD versus control group comparison with regard to the level of social media use, (b) a correlational analysis of the link between autistic traits and the level of social media use, and (c) a within-subjects comparison of social versus non-social media use in a sample of individuals with ASD. Thus, research that did not incorporate a control group, did not quantify autistic traits and/or social media use, or merely focused on the use of social media in ASD individuals (without paying attention to the employment of non-social digital media; e.g., Mazurek, 2013) were excluded from the meta-analysis (which again was performed using the JAMOVI software). Our predictions regarding the relationship between ASD/autistic traits and social media were less clear. As noted earlier, there are arguments to assume that individuals with ASD/high levels of autistic traits show less interest and involvement in social media (which would be reflected by a negative effect size), but it is also possible that individuals with ASD or high(er) on the autism spectrum (more often) engage socially on the internet as a compensation for the communication and interaction difficulties they experience in real life (Gillespie-Smith et al., 2021). Similar to the procedure that was followed for the meta-analysis of the relation between ASD/autistic traits and PIU, we carried out an extra search in the first week of 2025 to find new studies on the link between ASD/autistic traits and social media use that had been published in 2024.

The meta-analyses were not pre-registered. However, we closely followed the ‘Preferred Reporting Items for Systematic reviews and Meta-Analyses’ (PRISMA) guidelines (Page et al., 2021). Furthermore, a post-hoc evaluation by means of the ‘A MeaSurement Tool to Assess systematic Reviews’ (AMSTAR; Shea et al., 2007) indicated that our meta-analyses generally were of good quality: the research questions were well-defined, the literature search was extensive, clear inclusion criteria were employed (i.e., checks involving two raters of 12 included and 12 randomly selected excluded studies from each meta-analysis revealed a percentage of agreement of 91.67% (kappa = 0.83) in the case of studies on the relation between ASD/autistic traits and PIU, and 100% (kappa = 1.00) in the case of studies on the link between ASD/autistic traits and social media), the data extraction was straightforward, a well-known effect size calculator was used, and the likelihood of publication bias was evaluated through funnel plots.

Quality appraisals were made of the studies that were included in the meta-analysis using a combination of Murray et al.’s (2022a) criteria and the Journal Article Reporting Standards for quantitative research (Appelbaum et al., 2018). More specifically, studies on the relation between ASD/autistic traits and PIU were evaluated using the following five criteria: (1) Focus of the study and research question (minimal score = 0: constructs of ASD/autistic traits and PIU were neither included in the title of the paper nor in the research question(s) and hypotheses; maximal score = 2; both ASD/autistic traits and PIU were included in the title and the investigation of their relation was an explicit aim of the study); (2) Quality of the design (score = 1: study comparing PIU and non-PIU groups with regard to ASD diagnosis/autistic traits; score = 1.5: correlation analysis of the link between autistic traits and symptoms of PIU; score = 2: study comparing PIU between ASD and control group); (3) Adequacy of sample size (minimal score = 0: sample size < 50 for correlation analysis OR < 100 for both groups in PIU versus non-PIU comparison OR < 50 for both groups in ASD versus control comparison; maximal score = 2: sample size > 100 for correlational analysis OR > 100 for both groups in PIU versus non-PIU comparison OR > 50 for both groups in ASD versus control comparison); (4) Quality of the PIU measurement (minimal score = 0.5: use of a one-item scale; maximal score = 2: use of a standardized, psychometrically evaluated measure that was used by at least three different research sites); and (5) Quality of the ASD/autistic traits measurement (minimal score = 0.5: self-report diagnosis of ASD; maximal score = 2: clinical diagnosis supported by Autism Diagnostic Observation Schedule (ADOS; Lord et al., 2012) and ADI-R (Autism Diagnostic Interview-Revised; Lord et al., 1994) data.

For each study, a total score was calculated that was divided by the possible maximum of 10 to obtain a decimal score. Studies with a decimal score lower than 0.30 were classified as ‘low quality’, studies with a decimal score between 0.30 to 0.79 were defined as ‘moderate quality’, while studies with a decimal score of 0.80 or higher were considered as ‘high quality’ (Murray et al., 2022a). For the evaluation of the quality of studies on the relation between ASD and social media use, a comparable scoring procedure with similar criteria was employed. There was one exception: because the research field on social media use was less advanced, the criteria for rating the quality of the social media use measurement were somewhat more lenient (minimal score = 1: use of a one-item scale; maximal score = 2: use of a standardized, psychometrically evaluated measure). Quality ratings for all studies were made by the first author, after which the second author independently rated sets of 15 randomly selected studies on ASD/autistic traits and PIU and 15 randomly selected studies on ASD/autistic traits and social media use. The inter-rater (Pearson) correlations were 0.94 (*p* < 0.001) for studies on the relationship between ASD/autistic traits and PIU, and 0.87 (*p* < 0.001) for studies on the link between ASD/autistic traits and social media use. Furthermore, the quality ratings of the studies on ASD/autistic traits and PIU were also significantly correlated with those reported by Murray et al., (2022a; *k* = 20,* r* = 0.56, *p* = 0.01), although our criteria appeared to be somewhat more stringent, thereby yielding a lower overall quality score (average decimal score being 0.78 (*SD* = 0.09)—hence falling in the moderate quality range—versus 0.88 (*SD* = 0.08) as reported by Murray et al. (2022a)—indicating high quality [paired *t*(19) = 5.30, *p* < 0.001].

## Results

### ASD/Autistic Traits and PIU

**Fig. 1:**
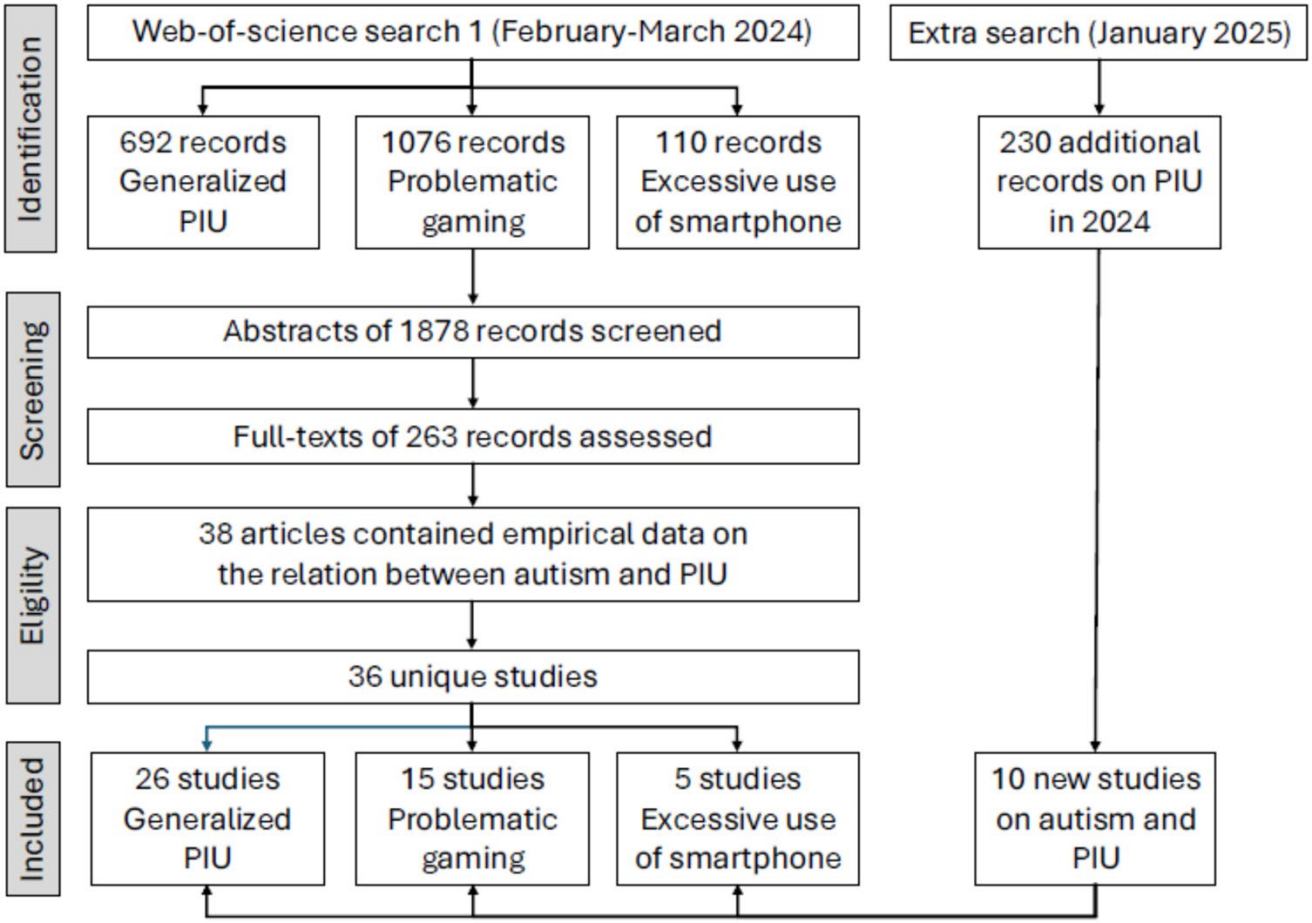
PRISMA flow diagram depicting the selection of articles that were included in the meta-analysis on the relations between ASD/autistic traits and PIU (left panel). *ASD* autism spectrum disorder, *PIU* problematic internet use

As can be seen in the PRISMA flow chart shown in Fig. 1, the initial literature search conducted in the early months of 2024 on the relation between ASD/autistic traits and PIU yielded 1878 results. After careful screening (and discarding two studies that presented additional or follow-up analyses of previously published data), eventually 36 unique studies were considered eligible and included in the meta-analysis. The extra search performed in January 2025 yielded 230 publications of studies published in 2024. These were carefully analyzed by means of our inclusion criteria. Ten new studies were found, resulting in a final number of 46 studies: 26 studies were concerned with generalized PIU, 15 with problematic gaming, and 5 with excessive smartphone use. The total number of participants included in all this research was 42,274. Samples consisted of children and adolescents (*k* = 20), young adults (with mean ages between 20 and 30 years; *k* = 18) and adults (with mean ages > 30 years; *k* = 8), who were either recruited from the general population (*k* = 26) or some clinical setting (*k* = 20). In terms of methodology, 23 studies employed a correlational design, 10 studies made a comparison between PIU and non-PIU groups, while 13 made a comparison between ASD and non-ASD-groups. Most studies employed self-report data for both PIU and ASD/autistic traits (*k* = 35); six studies used parent-report data for ASD/autistic traits, four studies solely relied on parent-report data, whereas one investigation obtained self- as well as parent-report data (see Table 1).

**Table 1:** Overview of studies examining the relationship between ASD/autistic traits and problematic internet use (PIU)

Study	Participants	Method	Type of PIU	Assessment PIU†	Assessment ASD/autistic traits
André et al. (2022) sample 1	8428 non-clinical adolescents aged 14–15 years (Sweden)	Comparison of groups with/without PIU	Problematic gaming	Excessive gaming (> 3 h per day)	Self-reported diagnosis of ASD
André et al. (2022) sample 2	7403 non-clinical adolescents aged 18–19 years (Sweden)	Comparison of groups with/without PIU	Problematic gaming	Excessive gaming (> 3 h per day)	Self-reported diagnosis of ASD
Arcelus et al. (2017)	245 adults (mean age = 27 years) referred to a transgender health service (United Kingdom)	Correlational	Problematic gaming	Internet Gaming Disorder Scale-Short Form^1^	Autism Spectrum Quotient^12^ (self-report)
Carpita et al. (2024a)	2574 university students (mean age = 25 years) (Italy)	Correlational	Generalized PIU	Assessment of Internet and Computer game Addiction^2^	Adult Autism Subthreshold Spectrum scale (self-report)^13^
Carpita et al. (2024b)	46 clinically referred adults with ASD (mean age = 34 years) and 53 healthy controls (Italy)	Comparison of groups with/without ASD	Generalized PIU	One-item measure of PIU	Adult Autism Subthreshold Spectrum scale (self-report)^13^
Charnock et al. (2024)	1178 non-clinical adults (mean age = 36 years) (United States of America, Australia, New Zealand)	Correlational	Problematic gaming	Internet Gaming Disorder checklist^4^	Ritvo Autism and Asperger Diagnostic Scale^14^
Chen et al. (2015)	1153 non-clinical children aged 7–14 years (Taiwan)	Comparison of groups with/without PIU	Generalized PIU	Chen Internet Addiction Scale^3^	Autism Spectrum Quotient^12^ (parent report)
Chou et al. (2017)	300 clinically referred adolescents with primary diagnosis of ADHD aged 11–18 years (Taiwan)	Comparison of groups with/without PIU	Generalized PIU	Chen Internet Addiction Scale^3^	Clinical diagnosis of ASD
Concerto et al. (2021)	4260 non-clinical adults aged 18–55 years who were members of online gaming community (multiple countries)	Correlational	Problematic gaming	Internet Gaming Disorder Scale-Short Form^1^	Autism Spectrum Quotient^12^ (self-report)
De Vries et al. (2018)	231 clinically referred adults aged 20–79 (Japan)	Comparison of groups with/without PIU	Generalized PIU	Internet Addiction Test^5^ and Compulsive Internet Use Scale^6^	Autism Spectrum Quotient^12^ (self-report)
Dell’Osso et al. (2019)	178 university students (mean age = 21 years) (Italy)	Comparison of groups with/without PIU	Generalized PIU	One-item measure covering excessive use and interference	Adult Autism Subthreshold Spectrum scale^13^ and Autism Spectrum Quotient^12^ (both self-report)
Engelhardt et al. (2017)	119 adults (mean age = 20 years) of whom half were recruited in a specialized ASD clinic and half from the general population (United States of America)	Comparison of groups with/without ASD	Problematic gaming	Pathological gaming measure	Clinical diagnosis of ASD (supported by ADI-R and ADOS)
Finkenauer et al. (2012)	195 married couples (*N* = 390; mean age = 31 years) recruited from the community (The Netherlands)	Correlational	Generalized PIU	Compulsive Internet Use Scale^6^	Autism Spectrum Quotient^12^ (self-report)
Fujiwara et al. (2018)	119 non-clinical adults (mean age = 35 years) (Japan)	Correlational	Generalized PIU	Generalized Problematic Internet Use Scale 2^7^	Autism Spectrum Quotient^12^ (self-report)
Hirota et al. (2021)	108 clinically referred adolescents with ASD and 3080 non-clinical adolescents from the community (aged 12–15 years) (Japan)	Comparison of groups with/without ASD	Generalized PIU	Internet Addiction Test^5^	Clinical diagnosis of ASD
Kawabe et al. (2019)	55 clinically referred children and adolescents with ASD aged 10–19 years (Japan)	Comparison of groups with/without PIU	Generalized PIU	Internet Addiction Test^5^	Autism Spectrum Quotient^12^ (parent-report)
Kawabe et al. (2022)	102 clinically referred adolescents aged 12–15 years of whom 35 were diagnosed with ASD (Japan)	Comparison of groups with/without ASD	Generalized PIU	Internet Addiction Test^5^	Clinical diagnosis of ASD (supported by ADI-R and ADOS)
Lee et al. (2022)	399 university students (mean age = 20 years) (Malaysia)	Correlational	Excessive smartphone use	Smartphone Addiction Scale^8^	Autism Spectrum Quotient^12^ (self-report)
Liu et al. (2017)	420 non-clinical children (mean age = 9 years) (China)	Correlational	Problematic gaming	Pathological Video Game Use Questionnaire^9^	Social and Communication Disorders Checklist^15^ (parent-report)
Lu et al. (2022)	1103 college students aged 18–26 years (China)	Correlational	Excessive smartphone use	Smartphone Addiction Scale^8^	Autism Spectrum Quotient^12^ (self-report)
Lyvers et al. (2024)	248 non-clinical adults aged 18–30 years (Australia)	Correlational	Generalized PIU	Internet Addiction Test^5^	Autism Spectrum Quotient^12^ (self-report)
MacMullin et al. (2016)	Parents of 172 typically developing individuals and 139 individuals with ASD aged 6–12 years (Canada and United States of America)	Comparison of groups with/without ASD	Generalized PIU and problematic gaming	Compulsive Internet Use Scale^6^ (parent-report)	Clinical diagnosis of ASD (supported by Social Communication Questionnaire^16^)
Masi et al. (2021)	101 clinically referred adolescents aged 11–16 years (Italy)	Comparison of groups with/without PIU	Generalized PIU	Internet Addiction Test^5^	Schedule for Affective Disorders and Schizophrenia for School-Aged Children^17^ (parent- and child-based interview)
Mazurek and Engelhardt (2013)	Parents of clinically referred boys with ASD (*n* = 56) or ADHD (*n* = 44), and typically developing boys (*n* = 41) aged 8–18 years (United States of America)	Comparison of groups with/without ASD	Problematic gaming	Problematic Video Gaming Test^10^ (parent-report)	Clinical diagnosis of ASD (supported by ADI-R and ADOS)
Mazurek and Wenstrup (2013)	Parents of 202 children and adolescents with ASD recruited via an Autism Network and typically developing youth (*n* = 179) aged 8–18 years (United States of America)	Comparison of groups with/without ASD	Problematic gaming	Problematic Video Gaming Test^10^(parent-report)	Parent-reported diagnosis (supported by Social Communication Questionnaire^16^)
Minami et al. (2024)	39 clinically referred adolescents with internet gaming disorder (mean age = 14 years) (Japan)	Correlational	Problematic gaming	Internet Addiction Test^5^	Autism Spectrum Quotient^12^ (self-report)
Murray et al. (2022b)	230 adults with ASD recruited via social media, support groups, and networks and 272 control participants (mean age = 30 years) (mainly Europe and United States of America)	Comparison of groups with/without ASD	Problematic gaming	Internet Gaming Disorder Test^11^	Autism Spectrum Quotient^12^ (self-report)
Ogawa et al. (2024)	399 college students (mean age = 19 years) (Japan)	Comparison of groups with elevated and normal levels of autistic traits	Generalized PIU	Internet Addiction Test^5^	Adult Autism Spectrum Disorder scale (self-report)^18^
Paulus et al. (2020)	Parents of 62 clinically referred boys with ASD and 31 typically developing boys aged 4–17 years (Germany)	Comparison of groups with/without ASD	Problematic gaming	Self-construed gaming disorder symptoms list (16 items) covering excessive use and interference (parent report)	Clinical diagnosis of ASD
Restrepo et al. (2020)	564 non-clinical children and adolescents aged 7–15 years and their parents (United States of America)	Comparison of groups with/without PIU	Generalized PIU	Internet Addiction Test^5^ (self- and parent-report)	Schedule for Affective Disorders and Schizophrenia for School-Aged Children^17^ (parent- and child-based interview) and other standardized scales including the Autism Spectrum Quotient^12^ (self-report)
Romano et al. (2013)	60 non-clinical adults (mean age = 24 years) (United Kingdom)	Correlational	Generalized PIU	Internet Addiction Test^5^	Autism Spectrum Quotient^12^ (self-report)
Romano et al. (2014)	90 non-clinical adults aged 20–30 years (United Kingdom)	Correlational	Generalized PIU	Internet Addiction Test^5^	Autism Spectrum Quotient^12^ (self-report)
Sahin and Usta (2020)	56 clinically referred adolescents with major depressive disorder aged 13–18 years (Turkey)	Correlational	Excessive smartphone use	Smartphone Addiction Scale^8^	Autism Spectrum Quotient^12^ (parent-report)
Shane-Simpson et al. (2016) study 1	597 college students aged 18–41 years (United States of America)	Correlational	Generalized PIU	Compulsive Internet Use Scale^6^	Autism Spectrum Quotient^12^ (self-report)
Shane-Simpson et al. (2016) study 2	33 college students with ASD recruited from a mentorship program and 33 typically developing students aged 18–37 years (United States of America)	Comparison of groups with/without ASD	Generalized PIU	Compulsive Internet Use Scale^6^	Self-reported diagnosis of ASD or clinical diagnosis
Simonelli et al. (2024)	77 clinically referred children and adolescents with ASD and 147 typically developing controls aged 8–17 years (Italy)	Comparison of groups with/without ASD	Problematic gaming	Internet Gaming Disorder Scale-Short Form^1^	Schedule for Affective Disorders and Schizophrenia for School-Aged Children^17^
Skotalczyk et al. (2024)	229 non-clinical adults aged 11–35 years (Poland)	Correlational	Generalized PIU	Internet Addiction Test^5^	Autism Spectrum Quotient^12^ (self-report)
So et al., (2017, 2019)	132 clinically referred adolescents with ASD and/or ADHD aged 12–15 years (Japan)	Comparison of groups with/without ASD (ADHD)	Generalized PIU	Internet Addiction Test^5^	Clinical diagnosis of ASD
Sulla et al. (2023)	141 university students aged 19–31 years (Italy)	Correlational	Generalized PIU	Internet Addiction Test^5^	Autism Spectrum Quotient^12^ (self-report)
Truzoli et al. (2019)	120 non-clinical adults aged 20–33 years (Italy)	Correlational	Generalized PIU	Internet Addiction Test^5^	Autism Spectrum Quotient^12^ (self-report)
Turan et al. (2024)	87 clinically referred adolescents with gaming-related problems and 83 typically developing controls aged 10–18 years (Turkey)	Comparison of groups with/without PIU	Problematic gaming	Internet Gaming Disorder Scale-Short Form^1^	Social Responsiveness Scale^19^ (self-report)
Umeda et al. (2021)	2227 non-clinical adults aged 20–75 years (Japan)	Comparison of groups with/without ASD	Generalized PIU	Internet Addiction Test^5^	Autism Spectrum Quotient^12^ (self-report)
Yang et al. (2024)	1107 non-clinical adults (partly students) aged 18–54 years (China)	Correlational	Excessive smartphone use	Smartphone Addiction Scale^8^	Autism Spectrum Quotient^12^ (self-report)
Zhang et al. (2021)	1524 non-clinical adults (mostly students) aged 18–82 years (China and Germany)	Correlational	Generalized PIU	Internet Addiction Test^5^	Autism Spectrum Quotient^12^ (self-report)
Zhang et al. (2022)	1103 non-clinical adults (mostly students) aged 18–59 years (China and Germany)	Correlational	Generalized PIU	Internet Addiction Test^5^	Autism Spectrum Quotient^12^ (self-report)
Zhou et al. (2024)	412 adolescent high school student aged 15–19 years (China)	Correlational	Excessive smartphone use	Smartphone Addiction Scale^8^	Autism Spectrum Quotient^12^ (self-report)

*ASD* autism spectrum disorder, *ADI-R* autism diagnostic interview-revised (Lord et al., 1994), *ADOS* autism diagnostic observation schedule (Lord et al., 2012)

^†^Unless otherwise indicated, PIU was assessed by means of a self-report measure

^1^Pontes and Griffiths (2015)

^2^Wölfling et al. (2010)

^3^Chen et al. (2013)

^4^Przybylski et al. (2017)

^5^Young (1998)

^6^Meerkerk et al. (2009)

^7^Caplan (2010)

^8^Kwon et al. (2013)

^9^Gentile (2009)

^10^King et al. (2011)

^11^Király et al. (2019)

^12^Baron-Cohen et al. (2001)

^13^Dell’Osso et al., (2017)

^14^Eriksson et al. (2013)

^15^Skuse et al. (2005)

^16^Rutter et al. (2003)

^17^Kaufman et al. (1997)

^18^Fukunishi (2016)

^19^Constantino (2002)

**Fig. 2:**
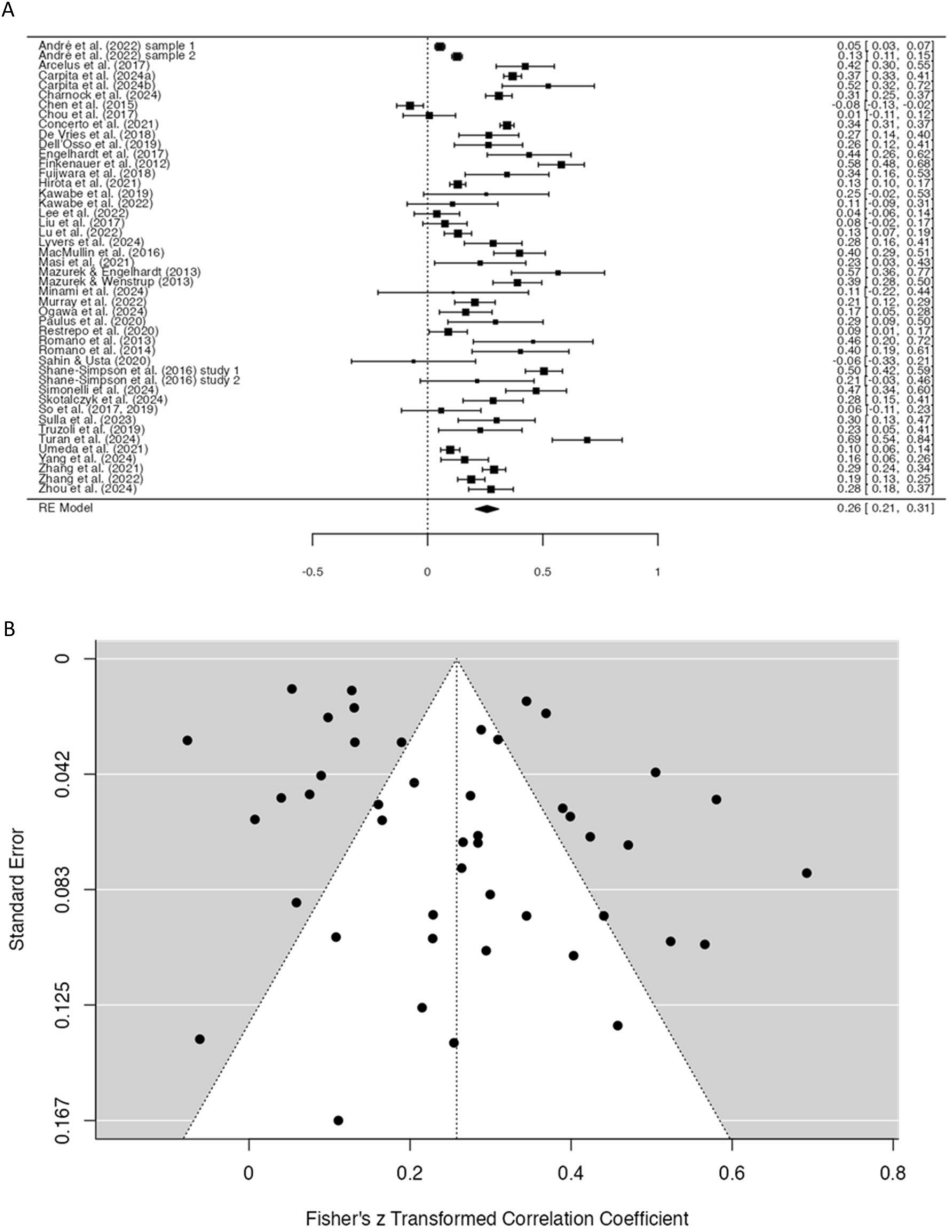
Forest plot (**A**) and funnel plot (**B**) of the meta-analysis on relationship between ASD/autistic traits and PIU

The effect sizes obtained in various studies can be seen in the forest plot shown in Fig. 2A. A medium overall effect size of 0.26 (*p* < 0.001) was found (with the 95% confidence interval (CI) ranging between 0.21 and 0.31), confirming the hypothesis that there indeed is a positive relationship between ASD/autistic traits and PIU. Using the binominal effect size display, an effect size of 0.26 suggests that when there are 100 people with ASD, 63 of them will have PIU compared to 37 without PIU. It should be noted that the data showed considerable heterogeneity (Q = 825.87, *p* < 0.001; I^2^ = 95.54%). Post-hoc analyses suggested that the magnitude of effect size was moderated by the type of PIU (i.e., average effect size was significantly larger for problematic gaming, *r* = 0.31, 95% CI [0.18, 0.43], than for generalized PIU, *r* = 0.24, 95% CI [0.10, 0.38] (*Z* = 6.36, *p* < 0.001), while the effect size for excessive smartphone use was even non-significant, *r* = 0.11, 95% CI [− 0.02, 0.23]), age group (i.e., effect size was significantly larger in young adults, *r* = 0.27, 95% CI [0.14, 0.40], and adults, *r* = 0.32, 95% CI [0.20, 0.45], than among children and adolescents, *r* = 0.20, 95% CI [0.05, 0.34], both *Z*’s ≥ 7.21, *p*’s < 0.001), and study method (i.e., effect size was significantly larger in correlational studies, *r* = 0.26, 95% CI [0.13, 0.39], and investigations making an ASD versus non-ASD group comparison, *r* = 0.28, 95% CI [0.13, 0.44], than in studies that made a comparison between PIU and non-PIU groups, *r* = 0.18, 95% CI [0.06, 0.30], both *Z*’s ≥ 7.73, *p*’s < 0.001). The type of population did not have a significant impact on the effect size of the relation between ASD/autistic traits and PIU (i.e., participants from general population, *r* = 0.24, 95% CI [0.13, 0.35] versus clinical participants, *r* = 0.26, 95% CI [0.10, 0.43]).

Inspection of the funnel plot (Fig. 2B) revealed that one third of the studies (66.67%) fell outside the 95% CI, again reflecting considerable variation in the effect sizes across the 46 studies (independent of the sample size and the accurateness of the obtained effect size). However, Egger’s test revealed no significant asymmetry (Egger’s test: *Z* = 1.37, *p* = 0.17), which suggests that data were not systematically biased (Egger et al., 1997).

As noted above, the overall quality of studies conducted to investigate the relationship between ASD/autistic traits and PIU was found to fall in the ‘moderate’ range (*M* = 0.78, *SD* = 0.09). This was mainly due to the lack of standardization in the assessment of ASD (*M* = 1.33 out of 2 possible points): 17 studies (36.96%) relied on a clinical diagnosis of ASD for which it was unclear how this had been established or even a self-reported diagnosis. Other less strong points of previous studies were the quality of the design (*M* = 1.54 out of 2 points; 21.73% of the studies was based on a comparison between PIU and non-PIU groups, which is a circumstantial way of investigating the link between ASD/autistic traits and PIU) and adequacy of the sample size (*M* = 1.52 out of 2 points; 50% of the studies relied on a suboptimal number of participants and hence were (potentially) underpowered).

## ASD/Autistic Traits and Social Media Use

**Fig. 3:**
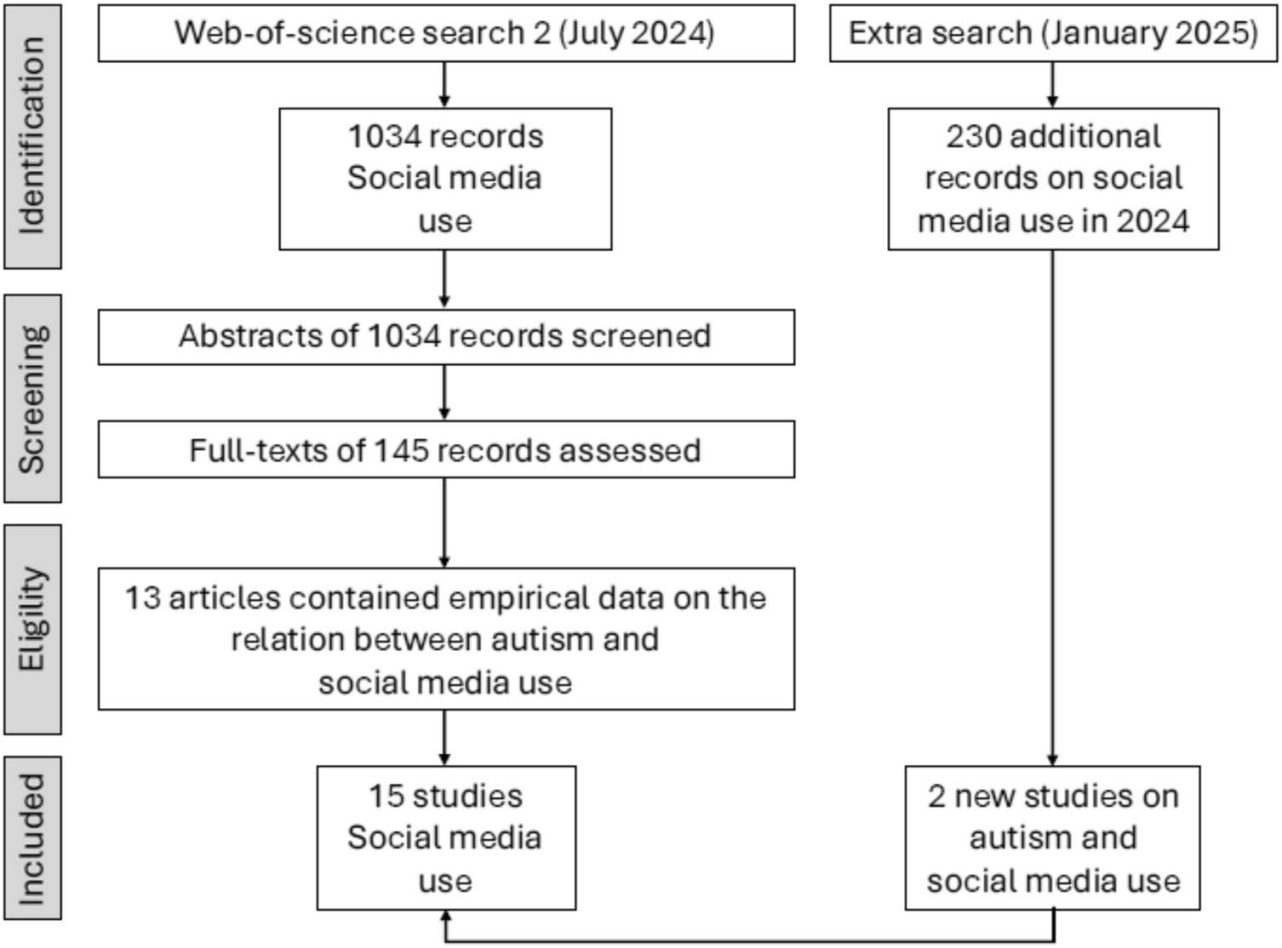
PRISMA flow diagram depicting the selection of articles that were included in the meta-analysis on the relations between ASD/autistic traits and social media use. *ASD* autism spectrum disorder

Figure 3 shows the search process of studies on the link between ASD/autistic traits and the use of social media. Eventually, 15 empirical studies that included a total of 7036 participants were detected and used as input for the meta-analysis. The details of these studies are reported in Table 2. Most studies (*k* = 10) compared the social media use between a group of participants with ASD and a group of typically developing people, one investigation made a within-subjects comparison of social versus non-social media use in a sample of participants with ASD, while four studies were correlational in nature and reported on the relationship between autistic traits and the frequency of social media use. Most studies included clinical participants (*k* = 13) and were focused on children and adolescents (*k* = 11).

**Table 2:** Overview of studies examining the relationship between ASD/autistic traits and social media use

Study	Participants	Method	Assessment social media^†^	Assessment ASD/autistic traits
Alhujaili et al. (2022)	26 clinically referred adolescents with ASD and 24 clinically referred controls aged 13–18 years (Canada)	Comparison of groups with/without ASD	Self-construed questionnaire to measure pattern and reasons for internet use: social versus non-social	Clinical diagnosis of ASD (supported by ADOS)
Begara Iglesias et al. (2019)	31 adolescents with ASD and 105 typically developing controls aged 10–25 years (Spain)^‡^	Comparison of groups with/without ASD	Self-construed questionnaire measuring use of digital devices and social media	Clinical diagnosis of ASD
Cardillo et al. (2025)	76 clinically referred adolescents with ASD and 107 typically developing controls aged 8–17 years (Italy)	Comparison of groups with/without ASD	Single item to measure frequency of social media use	Clinical diagnosis of ASD (supported by ADI-R)
Durkin et al. (2010)	35 adolescents with Asperger Syndrome and 35 typically developing controls aged 12–17 years (Australia)	Comparison of groups with/without ASD	Self-construed questionnaire to measure pattern and reasons for smartphone use: social versus non-social	Clinical diagnosis of ASD (supported by Childhood Asperger Syndrome Test^3^)
Kuo et al. (2014)	91 adolescents with ASD aged 12–18 years recruited via practitioners and school teachers (United States of America)	Within-subjects comparison of social versus non-social media use	Amount of time engaging in social versus non-social activities	Clinical diagnosis of ASD (supported by Social Communication Questionnaire^4^)
MacMullin et al. (2016)	Parents of 172 typically developing individuals and 139 individuals with ASD aged 6–12 years (Canada and United States of America)	Comparison of groups with/without ASD	Parent report of social media use	Clinical diagnosis of ASD (supported by Social Communication Questionnaire^4^)
Mazurek et al. (2012)	920 adolescents with ASD and 2590 adolescents with other neuro-developmental disorders aged 13–17 years who were receiving special education services (United States of America)	Comparison of groups with/without ASD	Parent report of social media use	Clinical diagnosis of ASD
Mazurek and Wenstrup (2013)	Parents of 202 children and adolescents with ASD recruited via an Autism Network and typically developing youth (*n* = 179) aged 8–18 years (United States of America)	Comparison of groups with/without ASD	Parent report of social media use	Parent-reported diagnosis (supported by Social Communication Questionnaire^4^)
Must et al. (2023)	186 children with ASD aged 9–10 years recruited via schools and 930 typically developing controls (United States of America)	Comparison of groups with/without ASD	Youth Screen Time Survey^1^: social activities	Parent-reported diagnosis of ASD
Paulus et al. (2020)	Parents of 62 clinically referred boys with ASD and 31 typically developing boys aged 4–17 years (Germany)	Comparison of groups with/without ASD	Parent-report of the child’s frequency of computer-mediated communication use	Clinical diagnosis of ASD
Sahin and Usta (2020)	56 clinically referred adolescents with major depressive disorder aged 13–18 years (Turkey)	Correlational	Social Media Disorder Scale^2^	Autism Spectrum Quotient^5^ (self-report)
Schuwerk et al. (2019)	234 non-clinical adults (mainly university students) aged 18–50 years (Germany)	Correlational	Frequency and duration of communication via smartphone	Autism Spectrum Quotient^5^ (self-report)
Suzuki et al. (2021) study 1	373 graduate and undergraduate students (mean age = 20 years) (Japan)	Correlational	Frequency and duration of social networking services (LINE) use	Autism Spectrum Quotient^5^ (self-report)
Suzuki et al. (2021) study 2	388 undergraduate students (mean age = 19 years) (Japan)	Correlational	Inactive use subscale of the self-construed LINE Use Questionnaire	Autism Spectrum Quotient^5^ (self-report
Van Schalkwyk et al. (2017)	44 clinically referred adolescents with ASD and 56 clinical controls aged 12–19 years (United States of America)	Comparison of groups with/without ASD	Self-reported use of social media	Clinical diagnosis of ASD (supported by ADI-R and ADOS)

*SD* autism spectrum disorder, *ADI-R* autism diagnostic interview (Lord et al., 1994), *ADOS* autism diagnostic observation schedule (Lord et al., 2012)

^†^Unless otherwise indicated, social media use was assessed by means of a self-report measure

^‡^This study also included a control group of children with intellectual disability (*n* = 45), but effect size obtained from this study was based on comparison between ASD and typically developing groups

^1^Bagot et al. (2022)

^2^Van denEijnden et al. (2016)

^3^Scott et al. (2002)

^4^Rutter et al. (2003)

^5^Baron-Cohen et al. (2001)

**Fig. 4:**
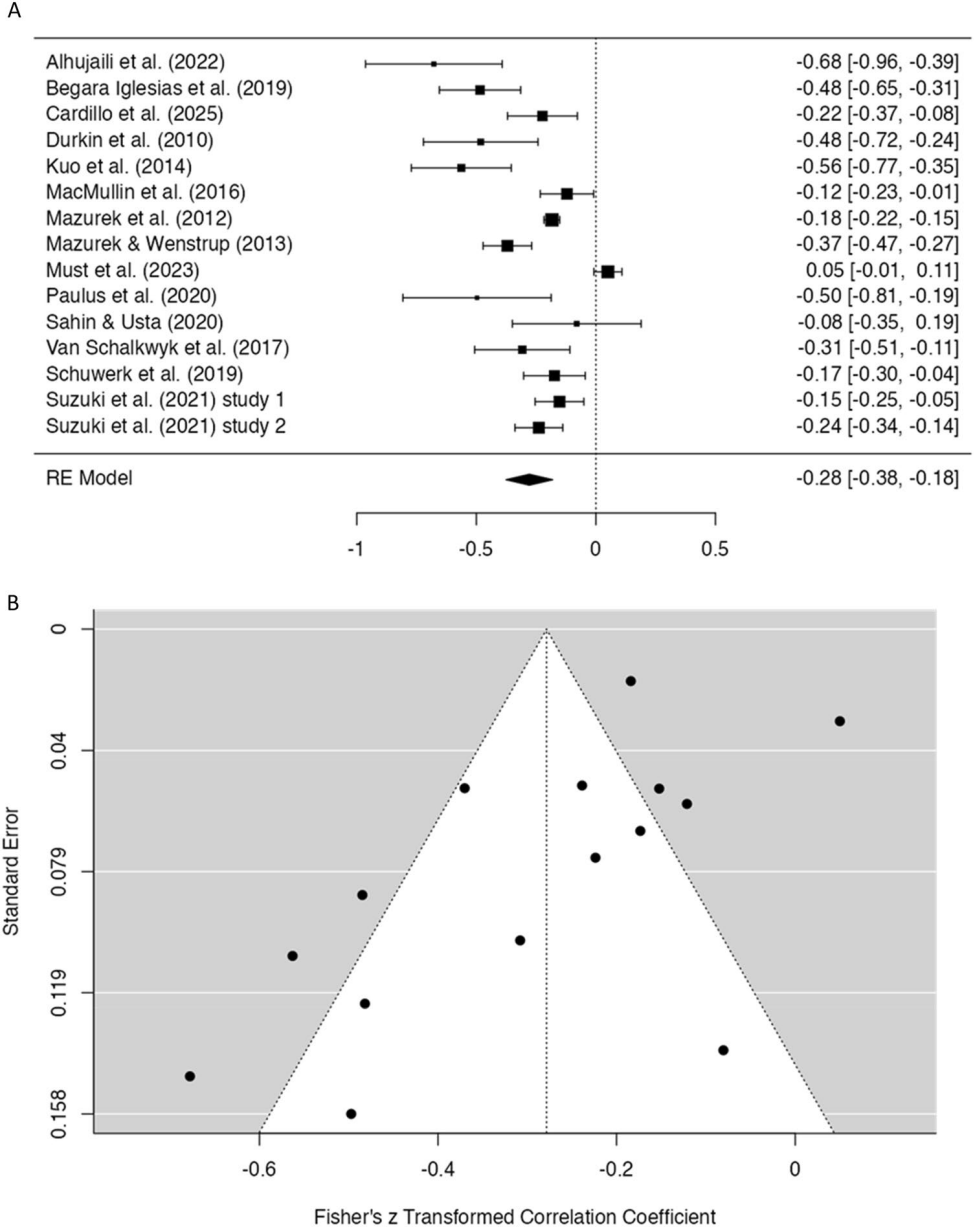
Forest plot (**A**) and funnel plot (**B**) of the meta-analysis on relationship between ASD/autistic traits and social media use

As can be seen in Fig. 4A, the forest plot showed that 13 out of 15 studies yielded a significant negative effect size, indicating that ASD/autistic traits were mostly associated with a lower frequency of social media use. The overall effect size was − 0.28, 95% CI [− 0.38, − 0.18], which also should be interpreted as a medium effect. The binominal effect size display indicated that 64 of every 100 people with ASD show a relatively low frequency of social media use compared to 36 people who exhibit a level of social media use that is comparable to individuals without ASD. Again, significant heterogeneity was noted (Q = 124.41, *p* < 0.001; I^2^ = 91.39%), but here no post-hoc analyses were conducted because the number of studies was limited and studies were more homogenous in terms of potential moderators (i.e., age group, study method, and type of population). The funnel plot (Fig. 4B) indicated that 46.67% of the studies were outside the 95% CI. Egger’s test was significant (*Z* = − 3.19, *p* = 0.001), which means that studies were not symmetrically located on the left and right side of the overall effect, possibly pointing at the presence of publication bias.

Ratings showed that the quality of the studies on the link between ASD/autistic traits and social media use could also be characterized as ‘moderate’. In particular, the assessment of ASD often lacked sophistication (*M* = 1.20 out of 2 points; 50.00% of the studies did not use a standardized measure to assess ASD) and this was also true for the assessment of social media use (*M* = 1.33 out of 2 points, in spite of more lenient criteria; 40.00% of the studies used a one-item scale). Furthermore, almost half of the studies (46.67%) appeared to rely on an inadequate sample size (*M* = 1.4 out of 2 points).

## Discussion

The results of our first meta-analysis confirmed the hypothesis that people with ASD or high levels of autistic traits indeed showed high levels of PIU (see also Craig et al., 2021; Eltahir et al., 2025; Murray et al., 2022a; Normand et al., 2022). However, there was considerable heterogeneity in effect sizes across studies, for which we were able to identify a number of potential moderators. First, effect sizes were stronger for studies that focused on problematic gaming than for studies that were concerned with generalized PIU, whereas studies on excessive smartphone use did not yield a significant effect size. Gaming is a circumscribed online activity that has inbuilt positive reinforcements, making this type of internet use highly ‘addictive’ (Kim et al., 2020) and this seems also true for individuals high on the ASD spectrum. In contrast, generalized PIU and excessive smartphone use are less well-defined and incorporate a much wider range of internet activities (some of which are problematic, while others are clearly not), and this may dampen the link with ASD/autistic traits. Second, studies comparing PIU and non-PIU groups were associated with smaller effect sizes. This was as anticipated: ASD/autistic traits constitute only one factor contributing to PIU, which is a phenomenon that may be associated with various other psychopathologies (e.g., ADHD, emotional disorders). Third, effect sizes for the relation between ASD/autistic traits and PIU were weaker in samples of children and adolescents than in samples of (young) adults, which could be explained by the influence of parents who during the childhood and teenage years to some extent can control the online behaviors of offspring thereby suppressing their level of internet use (Modecki et al., 2022).

The second meta-analysis demonstrated that persons with ASD or high levels of autistic traits are less inclined to use social media. Although they also appear to have a ‘need to belong’ (e.g., Deckers et al., 2014) and view digital social platforms as a feasible and perhaps more easy option to communicate and interact with others (Hudson et al., 2023), it is apparently not the case that people high on the autistic spectrum fully compensate their limited real-life socializing by social media activities. Note, however, that the number of studies on the relationship between ASD/autistic traits and social media use was limited, the effect sizes were quite heterogenous, and the possibility of publication bias could not be ruled out. Thus, to draw firm conclusions on the relation between ASD/autistic traits and social media use, more research is needed.

Quality appraisals revealed that the design and methodology of the research conducted on the relationship between ASD/autistic traits and PIU/social media use were in general of ‘moderate quality’. This is in line with Eltahir et al.’s (2025) conclusion on the quality of research on autism and gaming disorder and internet addiction (which was also typified as ‘moderate’), but somewhat at odds with Murray et al. (2022a) who gave a ‘high quality’ label to most studies in this research domain. Given that there was a clear positive correlation between our quality ratings and those provided by Murray et al. (2022a), the conclusion seems justified that our criteria for evaluating study quality (as well as those used by Eltahir et al., 2025) were somewhat more stringent.

In specific, we were more critical regarding the measurements that have been employed in this research. For example, to identify individuals with ASD, quite a number of studies relied on the clinical diagnosis of ASD, notwithstanding convincing evidence that inclusion of gold-standard instruments such as ADOS and ADI-R are necessary to firmly establish the presence of this disorder (Le Couteur et al., 2007). Studies measuring autistic traits oftentimes employ a shortened version of the AQ (Baron-Cohen et al., 2001), for which limited reliability and validity has been reported (Cheung et al., 2023). The assessment of PIU is mostly confined to self-report measures, which do not always give an accurate picture of people’s excessive online behavior (e.g., Babor et al., 1990). Importantly, it seems plausible to assume that underreporting of PIU is more likely to take place than overreporting, which would mean that the effect sizes obtained for many studies are an underrepresentation of the actual effects.

The measurement of social media use has also been a significant shortcoming of previous research. Studies typically do not use a standardized scale and simply focus on the frequency with which participants visit ‘social’ online platforms such as Facebook, Twitter/X, Instagram, and TikTok. Obviously, this yields a rather crude index of social media use that tells us little about how people engage in such platforms. Interestingly, Tuck and Thompson (2024) recently developed and validated the Social Media Use Scale, which evaluates social media use in terms of passive, active, social, and non-social categories, and hence might provide a more detailed picture of this type of online behavior.

Besides these measurement issues, studies on the relations between ASD/autistic traits and PIU/social media suffer from a number of other limitations. To begin with, studies that include persons with ASD typically focus on individuals who are on the high-functioning end of the spectrum, and so it remains largely unknown to what extent the current findings can be generalized to the internet use of autistic people with more severe symptoms and/or intellectual impairments. Furthermore, apart from a few exceptions (Liu et al., 2017; So et al., 2019), most studies were cross-sectional in nature. There is in general very little information on the developmental course of PIU (Huang et al., 2023), and virtually nothing is known about the progression of this type of problematic online behaviors in people with ASD (but see: Finkenauer et al., 2012). Finally, the research so far has not systematically looked at the possible role of gender differences. Meanwhile it is known that due to camouflaging, ASD symptomatology in girls/females is expressed in a different and often less severe way than in boys/males (Dean et al., 2017). In a similar vein, there are also sex differences in the manifestation of PIU and the use of social media (Mari et al., 2023), and thus it seems logical to include this demographic variable into account in future studies on the relations between ASD/autistic traits and PIU and social media use.

## Conclusions

Our meta-analytical approach confirms the results of previous systematic reviews (Craig et al., 2021; Eltahir et al., 2025; Murray et al., 2022a; Normand et al., 2022) showing that people with ASD or high levels of autistic traits generally display higher levels of PIU as compared to typically developing persons. Furthermore, persons with ASD generally displayed lower levels of social media use, which seems to be in line with the notion that individuals with this neurodevelopmental disorder have a diminished drive to engage socially than neurotypical people (Chevallier et al., 2012). However, one should be cautious with drawing a premature conclusion with regard to this point, because it may well be the case that persons with ASD do seek social interaction and communication on the internet, but rather use other digital resources (e.g., games) for this purpose instead of visiting the typical, common social media platforms (Hughes & Nguyen, 2024).

Research on the link between ASD and PIU could profit from studies conducted with greater methodological rigor (e.g., measuring PIU by other means than self-report, using gold standard instruments for assessing ASD and psychometrically sound versions of screening scales such as the full-length AQ to measure autistic traits), inclusion of low-functioning people with ASD, relying on longitudinal research designs, and taking into account gender differences). In addition, rather than focusing on global reports of PIU and social media use, it would be worthwhile to use ecological momentary assessment (EMA) to learn more about the link between this neurodevelopmental disorder and specific online behaviors. This method involves the repeated sampling of participants’ current behaviors, emotions, cognitions, and experiences in real time (Shiffman et al., 2008). Most importantly, the use of EMA would enable researchers to study the micro-processes that influence the online actions of people with ASD (and typically developing persons) in a real-world context. Such an approach could give a more precise picture of the internet activities that persons high on the autism spectrum engage in, how they maneuver on various digital media (e.g., engagement in chatting during gaming, social versus non-social and active versus passive use of social media)—thereby possibly revealing forms of idiosyncratic use, as well as gain more insight in the underlying motives of their online behavior. The motivation-based developmental psychopathology model that is described in Part II (Muris et al., 2025) provides valuable leads for designing future studies that aim to obtain greater insight in the online behaviors and mannerisms of people high on the autism spectrum.

## Data Availability

The Microsoft Excel-files containing the data of the meta-analyses can be obtained from the first author.
